# Spatial Proteomics toward Subcellular Resolution by Coupling Deep Ultraviolet Laser Ablation with Nanodroplet Sample Preparation

**DOI:** 10.1021/acsmeasuresciau.3c00033

**Published:** 2023-10-20

**Authors:** Piliang Xiang, Andrey Liyu, Yumi Kwon, Dehong Hu, Sarah M. Williams, Dušan Veličković, Lye Meng Markillie, William B. Chrisler, Ljiljana Paša-Tolić, Ying Zhu

**Affiliations:** † Environmental Molecular Sciences Laboratory, Pacific Northwest National Laboratory, Richland, Washington 99354, United States; ‡ Biological Sciences Division, Pacific Northwest National Laboratory, Richland, Washington 99354, United States; § Department of Microchemistry, Proteomics, Lipidomics and Next Generation Sequencing, Genentech, 1 DNA Way, South San Francisco, California 94080, United States

**Keywords:** spatial proteomics, deep ultraviolet laser ablation, nanoPOTS, proximity aerosol collection, subcellular resolution

## Abstract

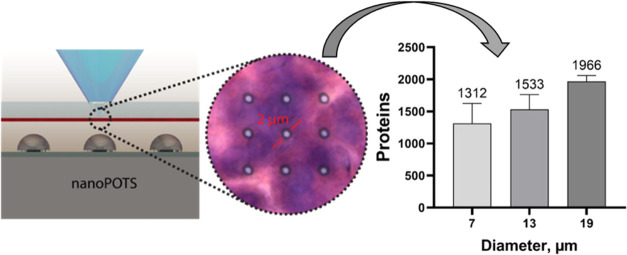

Multiplexed molecular profiling of tissue microenvironments, or spatial omics, can provide critical insights into cellular functions and disease pathology. The coupling of laser microdissection with mass spectrometry-based proteomics has enabled deep and unbiased mapping of >1000 proteins. However, the throughput of laser microdissection is often limited due to tedious two-step procedures, sequential laser cutting, and sample collection. The two-step procedure also hinders the further improvement of spatial resolution to <10 μm as needed for subcellular proteomics. Herein, we developed a high-throughput and high-resolution spatial proteomics platform by seamlessly coupling deep ultraviolet (DUV) laser ablation (LA) with nanoPOTS (Nanodroplet Processing in One pot for Trace Samples)-based sample preparation. We demonstrated the DUV-LA system can quickly isolate and collect tissue samples at a throughput of ∼30 spots/min and a spatial resolution down to 2 μm from a 10 μm thick human pancreas tissue section. To improve sample recovery, we developed a proximity aerosol collection approach by placing DMSO droplets close to LA spots. We demonstrated the DUV-LA-nanoPOTS platform can detect an average of 1312, 1533, and 1966 proteins from ablation spots with diameters of 7, 13, and 19 μm, respectively. In a proof-of-concept study, we isolated and profiled two distinct subcellular regions of the pancreas tissue revealed by hematoxylin and eosin (H&E) staining. Quantitative proteomics revealed proteins specifically enriched to subcellular compartments.

## Introduction

Multicellular species are highly complex containing multiple organs and tissues with heterogeneous cell populations.^1,2^ Each cell population has its distinct biological function, which is determined by the underlying bimolecular signatures. Indeed, recent developments in single-cell transcriptomics and proteomics technologies have revealed tremendous cellular diversity and largely extended our understanding of tissue and cell functions in both physiological and pathological environments. While single-cell technologies have been demonstrated to be valuable for understanding human disease by comparing the population changes during disease progression, it is still unclear how these increased or depleted cell populations impact tissue function.^2,3^ This can largely be attributed to tissue dissociation, which inevitably removes the critical spatial information on individual cells in native tissue. In a tissue environment, cells constantly communicate with each other by receptor/ligand binding or exchanging chemical factors. Thus, studying the cellular populations in native tissue microenvironments, or spatial omics, can provide critical insights into cell function, the origin and progression of disease, and potential mechanisms of drug resistance.^1,2,4−9^

Among the emerging spatial technologies, spatial proteomics provides the direct functional readouts of cellular/tissue phenotypes and thus has undergone rapid development in the past decade.^1,2,10−12^ Current spatial proteomics can be classified into two different approaches depending on whether an antibody is used or not. Antibody-based multiplex imaging approaches, such as CODEX (Co-detection by indexing),^13^ IBEX (Iterative bleaching extends multiplexity),^14^ IMC (Imaging Mass Cytometry),^15,16^ and MIBI (Multiplexed Ion Beam Imaging),^17^ have enabled mapping of >40 proteins on the same tissue samples at subcellular resolution. While these multiplex imaging approaches gained great interest, the multiplexity is limited. Additionally, protein quantification accuracy is largely determined by the affinity and specificity of antibodies. Antibody-free approaches allow for direct identification and quantification of proteins on tissue sections employing tissue microsampling followed by mass spectrometry (MS) to directly ionize the intact proteins or their peptides, followed by ion fragmentation and measurements.^18−20^ However, in these direct MS imaging approaches, the proteome coverage is typically below 100 targets primarily due to low-efficiency protein extraction as well as ionization suppression. Hence, only the most abundant proteins are typically detected.

MS-based bottom-up proteomics, where digested peptides were separated with liquid chromatography (LC), fragmented, and detected by MS, has become the gold standard of protein studies. With state-of-the-art LC-MS instrumentation, nearly the entire human proteome can be detected from cell and tissue specimens. The integration of microscale tissue sampling with LC-MS-based proteomics can provide the deepest coverage for spatial proteomics.^21−25^ Laser microdissection is the most widely used microsampling approach. It seamlessly couples high-resolution optical imaging microscopy with an infrared or ultraviolet laser to cut the region of interest from the tissue section and collect the pieces in capture devices. Indeed, recent studies demonstrated that thousands of proteins can be reliably mapped at a moderate spatial resolution of 100–200 μm^24,26,27^ and ∼1000 proteins at a high resolution of 20–50 μm.^28^ Such a deep proteome coverage allows the characterization of key signaling proteins within the tissue microenvironment.

The ultimate goal of spatial proteomics is to achieve large-scale mapping of proteins with high spatial resolution and high throughput. Recently, the throughput of LC-MS-based proteomics has been significantly improved with advanced LC systems,^29,30^ sample multiplexing, and data-independent acquisition (DIA) MS methods.^31^ Artificial-intelligence-driven image analysis algorithm enabled automated cell segmentation and selection, boosting the speed of feature selection for spatial proteomics.^28^ Despite these advances, the overall throughput remains limited primarily by a two-step laser dissection process. Additionally, the laser cut surrounding ROI limits the achievable spatial resolution to >10 μm because the minimal size of ROI is 4-fold larger than the width of the laser cutting line. Additionally, conventional laser microdissection requires the use of polymer membrane-coated slides, which is associated with several technical challenges including poor adhesion of tissue, significant autofluorescence background, low optical transparency, and reduced protein extraction efficiency.

To address these challenges, we coupled deep ultraviolet (DUV) laser ablation (LA) with a nanodroplet-based sample preparation system to advance both throughput and resolution of spatial proteomics. DUV-LA enables protein sampling from the tissue in a single step, thus significantly increasing the sampling speed. Compared with other lasers, DUV can be focused on a smaller spot, which is critical for high-resolution spatial sampling. DUV-LA does not use membrane-coated slides and therefore enables high-quality optical tissue imaging, which is critical for feature detection and automated cell segmentation. DUV-LA has been widely used to perform MS tissue imaging for small molecules and proteins.^32^ For example, DUV-LA coupled with inductively coupled plasma mass spectrometry (ICP-MS), or mass cytometry imaging, has enabled high-plex protein mapping at a spatial resolution of ∼2 μm.^15^ Recently, the Murray group^33^ integrated DUV-LA with MS-based spatial proteomics and demonstrated high tissue sampling efficiency. Their study also indicated that DUV-LA can generate smaller tissue particles without causing protein fragmentation.^34^ Although this study demonstrated the feasibility of DUV-LA for spatial proteomics, both the spatial resolution and proteome coverage were moderate,^33^ likely due to the low protein recovery with the conventional tube-based sample preparation approach. By coupling DUV-LA with nanoPOTS (Nanodroplet Processing in One pot for Trace Samples), we significantly improved sensitivity and consequently proteome coverage. We designed and assembled a fully automated optical and robotic system to achieve microscopic imaging, cell selection, DUV-LA triggering, and sample collection into a nanodroplet. We developed a proximity aerosol collection (PAC) approach to maximize the sample recovery during LA. Systematic optimization of the DUV-LA-nanoPOTS platform allowed for tissue sampling at ∼2 μm resolutions and mapping of >1300 proteins at ∼7 μm resolution.

## Experimental Section

### Reagents and Chemicals

Deionized water (18.2 MΩ) was prepared in a Barnstead Nanopure Infinity system (Los Angeles, CA) and used throughout. Acetonitrile (ACN), ATTO-Tag-FQ amine derivatization kit, borax stock buffer, chloroacetamide (CAA), dithiothreitol (DTT), formic acid, *n*-dodecy-ß-d-maltoside (DDM), iodoacetamide (IAA), MeOH, and (tris(2-carboxyethyl)phosphine)-HCl (TCEP) were purchased from Thermo Fisher Scientific (Waltham, MA). 1H,1H,2H,2H-perfluorododecyltrichlorosilane, 4-(2-hydroxyethyl)-1-piperazineethanesulfonic acid (HEPES, pH 8.5), and toluene were purchased from Sigma-Aldrich (St. Louis, MO). LysC and trypsin were purchased from Promega (Madison, WI).

### NanoPOTS Chip Fabrication

The fabrication of the 5 × 13 (row × column) nanoPOTS was based on a protocol derived from our previous study.^22^ Briefly, starting with a glass slide with precoated Chrome (Cr) and photoresist (Telic company, Valencia, CA), standard photolithography and wet etching were used to generate the nanowell pattern. After development and Cr etching, 5 × 13 pedestals (wells) formed with Cr covered on them. The exposed surface surrounding the 1 mm diameter pedestals was rendered hydrophobic by treating with 1% 1H,1H,2H,2H-perfluorododecyltrichlorosilane in toluene at room temperature for 1 h. The hydrophilic surface on the pedestals was revealed by removing the Cr layer on them with a chromium etching solution. Finally, the nanoPOTS chips were cleaned thoroughly with a detergent solution and nanopure water. Prior to sample collection, all nanowells were loaded with 150 nL of DMSO droplets.

### Design and Assembly of the DUV-LA-nanoPOTS Platform

**Figure 1:**
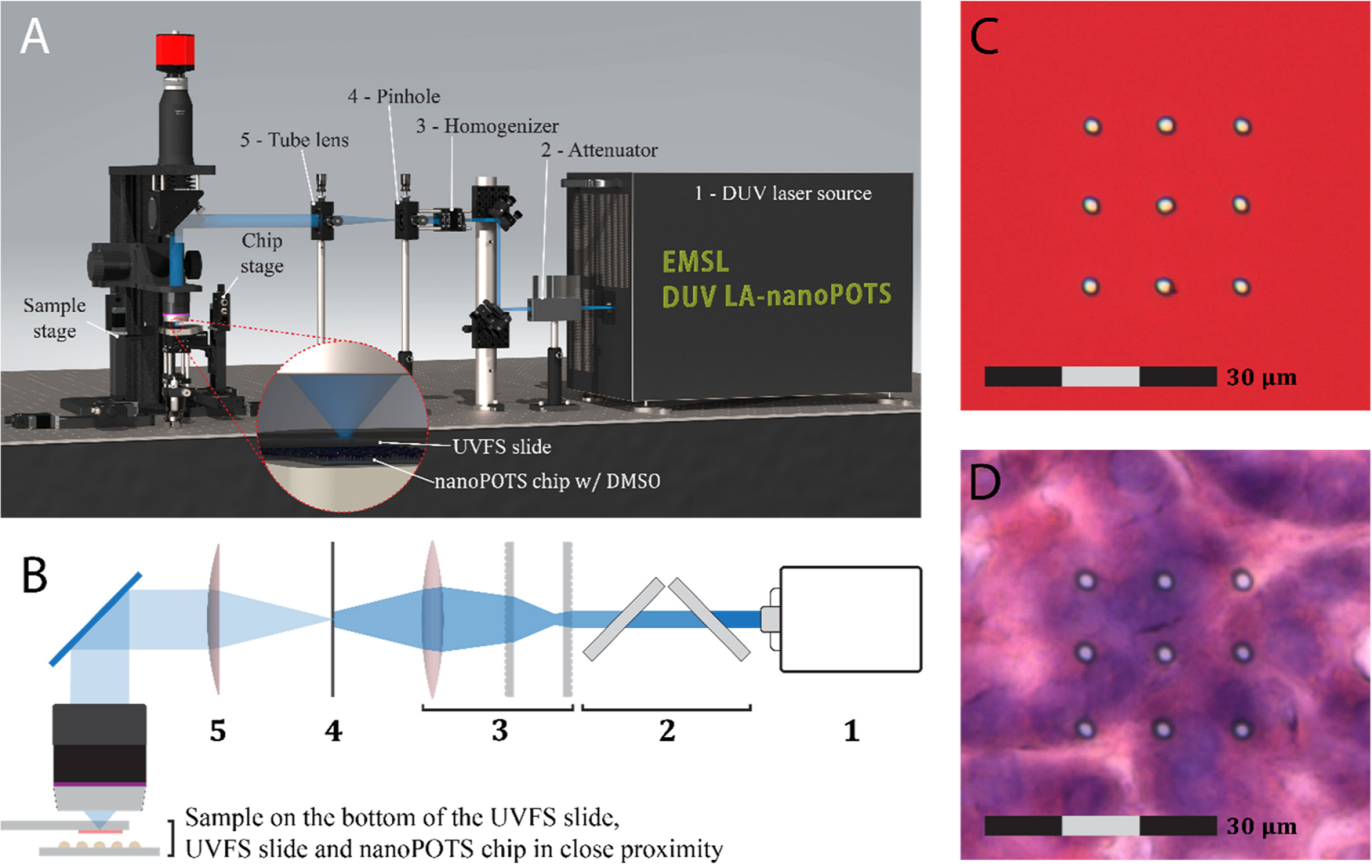
(A) Rendered three-dimensional image of deep ultraviolet (DUV) laser ablation (LA) system coupled with a nanoPOTS device for high-resolution spatial proteomics. (B) Schematic diagram of the DUV optical components. The name of each component was indicated in (A) with the same index number. (C) Photoimages of DUV ablated spots on a Sharpie dye-coated slide and (D) H&E stained pancreas section (10 μm thickness). Both (C,D) are 3 × 3 arrays (array distance of 10 μm) ablations with a spot diameter of ∼2 μm.

A rendered image of the platform is shown in Figure 1A. It consists of a mechanical system, an optical system (Figure 1B), and a distance measurement system (Figure S1). The mechanical system has two separate motorized XYZ stages (Zaber, Vancouver, BC, Canada) that control the movement of the sample slide and nanoPOTS chip independently. The sample slide mount and chip holder were 3D printed and connected to their corresponding XYZ stages via kinematic bases (Thorlabs, Newton, NJ) for easy attachment/detachment. For the optical system, a Coherent excimer laser (ExciStar 200, 193 nm, Santa Clara, CA) was employed as the DUV laser source. A 3D-printed holder with attenuator slides (regular quartz slides with significant absorption at 193 nm, Ted Pella, Redding, CA) was used to adjust the laser energy. A microlens array (Newport, Irvine, CA) was assembled to homogenize the laser spot. A tungsten-foil pinhole (Thorlabs, Newton, NJ) was placed on the homogenization plane. A tube lens (*f* = 200 mm, Thorlabs, Newton, NJ) was used for collimation and a reflective objective (20×/0.33, ∞/0, Edmund Optics, Barrington, NJ) was used to focus the laser onto the sample. A customized condenser light source was placed under the objective. The reflective objective, an *f* = 200 mm tube lens (Thorlabs, Newton, NJ), and a color CCD camera (Thorlabs, Newton, NJ) also constitute a coaxial image acquisition system. A Dino-Lite camera mounted on a manual XYZ translation stage and a second light source (Figure S1) were used to measure the collection distance. A Labview program (Figure S2) was developed to control the color CCD camera, the two motorized XYZ stages, and to send triggers to the DUV laser source. The Dino-Lite camera was controlled by the manufacturer’s software.

### Alignment of the Laser Focus Point and nanoPOTS Chip

Due to the low energy required to ablate the dye, the lowest constant energy output (2 mJ) was set on the DUV laser system and six pieces of attenuation slides were installed. A UV-grade Fused Silica (UVFS) slide (AdValue Technology, Tucson, AZ) coated with Sharpie dye was installed on the sample stage. The sample Z stage was adjusted to obtain a clear image of the UVFS slide on the dye side. One laser pulse was emitted. The LA spot location was adjusted by changing the angle of the laser reflectors until it was around the center of the camera window. The location of the ablation spot on the image was manually recorded in the LabVIEW software. An on-screen marker was also placed on the ablation spot. The dye-coated slide was then detached from the sample XYZ stage. A nanoPOTS chip was attached to the chip’s XYZ stage. A parking position of the nanoPOTS chip was set. The chip XYZ stage was moved until the left-rear alignment spot (Figure S3) was visible and focused in the LabVIEW camera window. Then, it was carefully adjusted until the center of the alignment spot overlapped the on-screen marker. The left-front and right-front alignment dots were aligned with a similar method sequentially. The fourth alignment spot (right back) was used to check if the previous three alignment spots were set correctly. Finally, nanoPOTS was moved to the parking position.

### Optimization of Collection Distance

**Figure 2:**
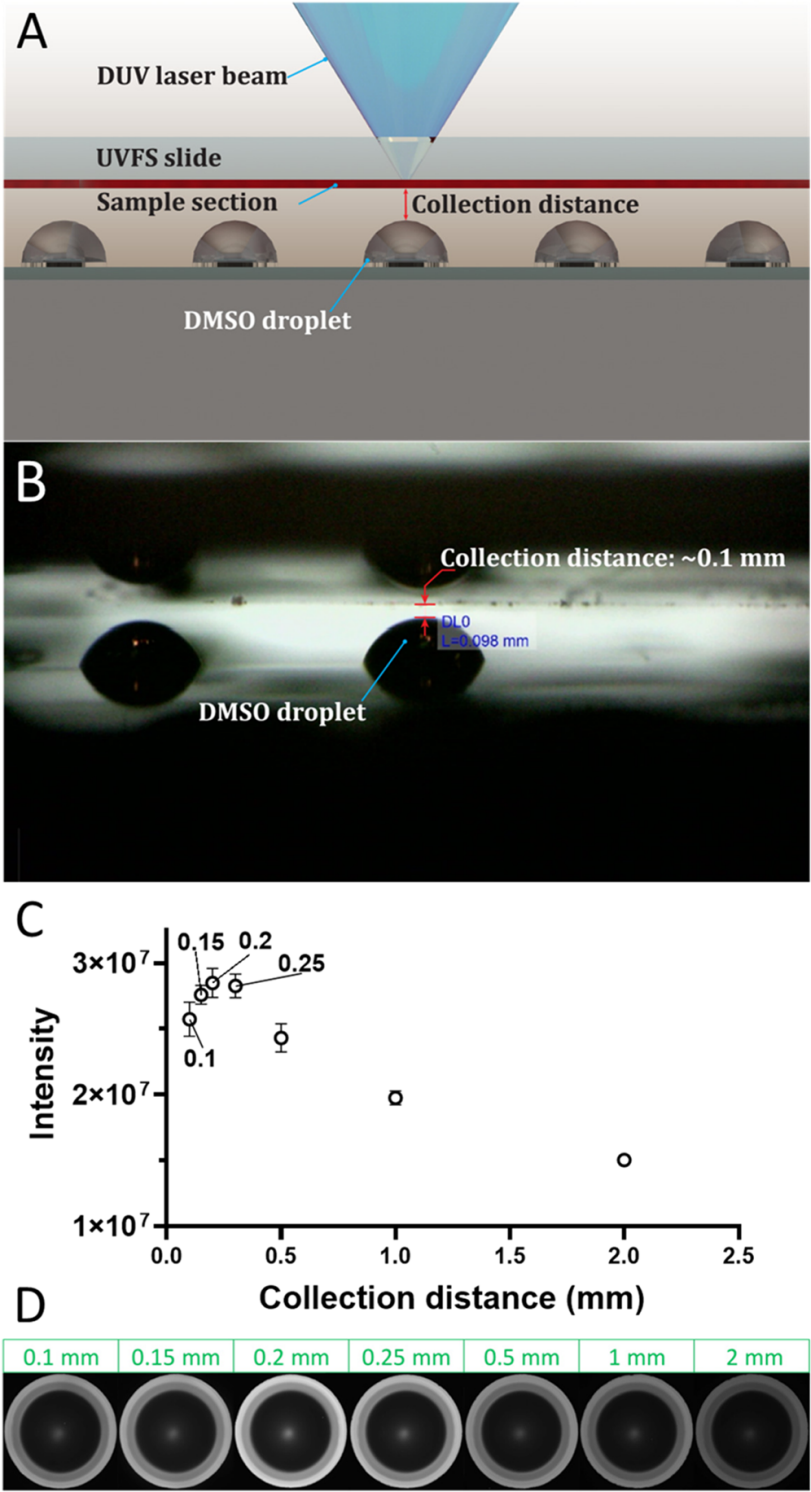
(A) Schematic diagram of a UVFS slide and closely placed nanoPOTS chip with preloaded DMSO droplets during sample collection. (B) Photo image indicating a collection distance of ∼0.1 mm. (C) Relationship between collection distance and collected protein amounts from a 25 μm diameter spot. The collected protein amounts were quantified with a fluorescent assay indicated in Experimental Section. (D) Fluorescence images of nanoPOTS wells at different collection distances.

We define the collection distance as the gap between the top of DMSO droplet used for collecting sample aerosol and the sample slide directly below the objective (Figure 2A,B). To optimize the collection distance, we adjusted the laser beam to create a single shot with a relatively large circular area (∼25 μm in diameter) to minimize discrepancies from the sample heterogeneity. Before sample collection, the laser spot shift and chip alignment were adjusted as described above. The sample slide and the nanoPOTS chip were moved to the collection position with the desired collection distance, which was achieved by applying a delta Z to the designated well’s normal Z position (Figures S3, S4). The Dino-lite camera was used to measure the collection distance (Figure 2B). One laser shot was fired at each spot. After a ∼5 s delay, the sample slide was moved to a new region and the nanoPOTS chip was moved to another well. Five replicates were performed for each collection distance. Immediately after sample collection, the nanoPOTS chip was covered and transferred to the home-built robotic liquid handling system for adding fluorescence labeling reagent at 150 nL per well. The fluorescence labeling reagent consisted of a 1:1 (vol) mixture of 5 mM ATTO-Tag-FQ in MeOH and 10 mM KCN in 50 mM borax buffer. The chip was then covered and wrapped with aluminum foil and placed at room temperature for 1 h. A fluorescence microscope (Zeiss PALM Microbeam, Munich, Germany) was used to capture the images of the wells. The fluorescence intensity of each nanowell was analyzed with ImageJ.

### Operation of the DUV-LA-nanoPOTS Platform for Sample Collection

A nanoPOTS chip preloaded with DMSO was aligned as described above. Next, the chip was moved to the parking position, and the UVFS slide with tissue section was installed on the sample stage. The distance between the sample slide and the DMSO droplet was adjusted with the distance measurement system (Figure S1). The chip was moved to the parking position again. A 3 × 3 image grid (Figure S5) of the tissue section containing the region of interest was acquired with the software. The image tiles had a ∼4% overlap in both the horizontal and vertical directions. ImageJ was used to stitch the images and generate image pixel coordinate information. LabVIEW software utilized the sample-XYZ-stage position of each image tile and the pixel coordinate information to generate a pixel-to-stage position conversion matrix. Regions of interest (ROI) were selected with their receiving wells’ position (row/column) specified. Additional parameters, such as laser pulse frequency and cut speed, were set or modified if necessary. Once the sample collection was complete, the sample ROIs were inspected using the optical imaging system (Figure 1B).

### Pancreas Section Preparation

Optimal cutting temperature compound (OCT) embedded human pancreas tissue block was obtained from the University of Florida as a part of the HuBMAP project. The tissue block was sectioned using cryotome at 10 μm thickness and mounted on UVFS slides. The pancreas section was fixed with 70% ethanol (EtOH), followed by a standard H&E staining protocol to visualize the tissue.^27^ Next, the tissue sections were dehydrated with 95 and 100% ethanol. Finally, the tissue slides were dried, vacuum sealed, and stored at –20 °C until use.

### Pancreas Library Sample Preparation

The library sample was generated by using an adjacent tissue block. The tissue was sectioned at 100 μm thickness, and the tissue rolls were collected in an Eppendorf tube. The sample was fixed by incubating in 75% EtOH at room temperature on a shaker at 500 rpm for 5 min. EtOH was removed and replaced with the Milli-Q deionized water. The sample tissue was shaken with the same settings. The water washing was repeated one more time to remove the OCT polymer. The sample was homogenized with a pellet pestle in 150 μL of 50 mM NH_4_HCO_3_ buffer containing 8 M urea. A bicinchoninic acid (BCA) assay was performed to determine the protein concentration. Protein reduction was performed by adding DTT to achieve a final concentration of 5 mM and incubating on a shaker at 850 rpm and 37 °C for 1 h. Next, proteins were alkylated by 15 mM IAA for 1 h in the dark. The sample was diluted 8× with 50 mM NH_4_HCO_3_ and with 1 M CaCl_2_ to have a final concentration of 1 mM CaCl_2_. Trypsin was added to the sample in a 1:20 (trypsin/protein) weight ratio. The sample was digested at 37 °C for 3 h on a shaker at 850 rpm. After digestion, the sample was cleaned up with an SPE column.

### Proteomic Sample Preparation with nanoPOTS

Label-free sample preparation followed an established nanoPOTS protocol.^26,35^ Briefly, DMSO droplets on nanoPOTS chips were evaporated by placing the chip in an oven at 70 °C for 30 min. A home-built robotic liquid handling system was used to deliver reagents to wells. Proteins were extracted and reduced by adding 150 nL of extraction buffer (1 mM TCEP, 0.1% DDM, and 0.1 M HEPES) and incubated in an oven at 70 °C for 1 h. Next, 50 nL of alkylation buffer (10 mM CAA in 0.1 M HEPES) was added to each well and incubated in the dark at room temperature for 30 min. Next, 50 nL of enzymatic digestion buffer (0.01 ng·nL^–1^ LysC and 0.04 ng·nL^–1^ Trypsin in 0.1 M HEPES) was added and incubated at 37 °C for 10 h. The reaction was quenched by adding 50 nL of 5% formic acid. Finally, the droplets were evaporated inside a vacuum desiccator, and the chip was stored at −20 °C until analysis.

### LC-MS/MS Analysis

LC-MS analysis was performed with a home-built nanoPOTS LC and a Tribrid Lumos Orbitrap MS with a FAIMSpro interface.^36^ Briefly, the nanoPOTS LC instrument was used to perform sample extraction/injection, solid phase extraction (SPE) cleanup, and LC separation automatically. Both the SPE column (100 μm i.d., 4 cm, 5 μm C18 packing material (300 Å pore size; Phenomenex, Torrance, CA)) and nanoLC column (50 μm i.d., 25 cm long, 1.7 μm, C18 packing material (BEH 130 Å C18 materials, Waters, Milford, MA)) were packed in-house. The LC column was heated at 50 °C with a column heater (Analytical Sales and Services Inc., Flanders, NJ) and operated at 100 nL/min. A 30 min linear gradient from 8 to 22% buffer B (0.1% FA in ACN) followed by a 9 min linear gradient from 22 to 35% buffer B was used.

For MS data collection, the electrospray ionization voltage was set at 2.4 kV. Three FAIMS compensation voltages (CVs) including −45, −60, and −75 V were used at 0.8 s per cycle. At each CV cycle, MS1 acquisition was performed at a resolution of 120 k, an AGC of 1E6, and a maximum injection time (IT) of 500 ms. For MS/MS acquisition, precursors with intensities >1E4 were isolated with a window of 1.4 *m*/*z*, an AGC of 2E4, and an IT of 150 ms. The precursor ions were fragmented by 30% HCD and detected in an ion trap. For generating the spectra library, each 50 ng of peptide was analyzed using a single CV at a time. The cycle time was set at 2 s. MS1 acquisition was performed at an IT of 118 ms. For MS/MS acquisition, precursor ions were isolated with an IT of 86 ms. Other parameters were the same as those described above.

### Data Analysis

The MS data were analyzed with transferring identification based on the FAIMS filtering (TIFF) as previously reported.^35^ Briefly, a reference library was constructed with separate analyses at each compensation voltage (CV). Low-input samples were cycled through multiple FAIMS CVs within a single LC-MS analysis. Peptides were identified by matching to the reference library using LC retention time, accurate *m*/*z*, and FAIMS CV. All data were processed with FragPipe (Ver. 19.2)^37^ and IonQuant (Ver. 1.9.2)^38^ with an activated match between runs (MBR) algorithm. The UniProt protein sequence database had decoy sequences (Proteome ID: UP000005640; downloaded on 04/10/2023, containing 20455 reviewed sequences). The search parameters were set as follows: a precursor mass tolerance of ±20 ppm, fragment mass tolerance of ±20 ppm, strict trypsin as the cleavage enzyme, carbamidomethylation of cysteine as a fixed modification, and oxidation of methionine as variable modification. Both protein and peptide identifications were filtered to a false discovery rate of <0.01 within MSfragger. For MBR, a false discovery rate of 0.05 was applied at the ion level. Data cleanup, normalization, statistical analysis, and visualization were performed with Perseus and PRISM.

### Data Availability

The mass spectrometry raw data have been deposited to the ProteomeXchange Consortium via the MassIVE partner repository with data set identifier MSV000086809 and are available at ftp://massive.ucsd.edu/MSV000086809/ (FTP server: massive.ucsd.edu; User name: MSV000092014.

## Results and Discussion

### Development of the DUV-LA-nanoPOTS Platform

We developed a fully automated DUV-LA system and coupled it with a highly sensitive nanoPOTS sample processing system and a state-of-the-art LC-MS platform. We reason that the DUV-LA-based sample isolation could significantly improve the performance of spatial proteomics. First, LA directly generates samples in a single-step aerosolization process at a speed of milliseconds per spot. In this proof-of-concept study, a sample collection throughput of 30 samples (spots) per minute was achieved through coupling of DUV-LA with robotic stages; the use of higher-speed stages is expected to further improve performance. Second, the minimal size of the ROI is equivalent to the size of the LA spot. Under optimized conditions, a spot diameter of ∼2 μm can be readily achieved on both a dye-coated slide and a 10 μm pancreas tissue section (Figure 1C,D). Importantly, the ablation spot shape and size can be tuned by adjusting the pinhole size and laser energy level to meet the requirements of different applications. For example, Figure S6 shows a square ablation spot obtained using a square pinhole. Third, LA directly transforms solid tissue into micrometer-sized particles, which could improve the protein extraction efficiency and consequently the overall proteome coverage. Finally, the DUV-LA system enables the use of a fused silica slide without polymer membrane coating. Thus, high-quality optical images of tissue sections can be readily obtained.

We made several key improvements in optical design to achieve a reliable and reproducible LA process (Figure 1A,B). We employed an array of quartz slides to attenuate the laser output energy. For a 193 nm excimer laser, ∼30% energy can be absorbed by a single regular quartz slide. Together with the pinhole, the laser output energy can be reduced from mW to nW scale. The original laser beam has a rectangular shape with a Gaussian intensity profile, where the spot center has the highest energy. Thus, ablations with the original laser beam often produce oval-shaped spots. To generate a homogeneous laser beam at the focal plane, where the pinhole was positioned, we employed a microlens array and positioned a tube lens behind the pinhole for collimation. A reflective objective was used to replace commonly used refractive objective to achieve both highly efficient focusing of 193 nm excimer laser and high-resolution optical imaging with visible light.

### Proximity Aerosol Collection for Maximal Collection Efficiency

The key difference between LA and LCM is the aerosolization process, which generates nanometer- and micrometer-sized particles. After aerosolization, the particles are ejected at high speed but quickly decelerate in ambient conditions and can quickly dissipate if they are not collected promptly and efficiently. To address this challenge and maximize sample recovery, we developed a proximity aerosol collection (PAC) approach. We reason that the particles should be collected as soon as they are generated and before they dissipate in air. To accomplish that, we positioned DMSO droplets^27^ preloaded on nanowells in proximity to the LA spot to capture the aerosolized particles efficiently. The distance between the tissue section and the DMSO droplet was critical for achieving high collection efficiency. To assess its impact, we collected samples with collection distances of 0.1 0.15, 0.2, 0.25, 0.5, 1, and 2 mm and measured the total protein amount of each sample with a fluorescent assay. As shown in Figure 2C,D, the fluorescent intensities increased as the distance increased from 0.1 to 0.2 mm, then quickly decreased starting at 0.25 mm. The highest intensity at 0.2 mm was ∼1.7E7, while the intensity at 2 mm was only ∼3E6, indicating that <17.6% of protein particles were collected at 2 mm.

We hypothesized that the significantly reduced velocity and disperse angle of the aerosol stream cause low efficiency at large collection distances. Surprisingly, we also observed decreased fluorescence intensity at small collection distances of <0.2 mm. This is likely caused by DMSO splashing because of the strong impact of high-velocity aerosol particles on the droplet surface. As shown in Figure S7, the splashed DMSO droplets carrying sample particles were deposited back on the tissue section at a collection distance of 0.1 mm. To test this hypothesis, we ablated pancreatic tissue at different energy outputs. As expected, higher energy led to more severe DMSO splashing (Figure S8). Another possible explanation is that the DMSO splash may be caused by UV LA itself. To investigate this, we repeated ablation on the region of the slide without the sample. Even at the highest energy output, no DMSO splashing was observed (Figure S8). Ultimately, a collection distance of 0.2 mm was confirmed to be the optimal distance for a most efficient and consistent sample collection.

### Evaluation of Potential Protein Fragmentation and Oxidation

**Figure 3:**
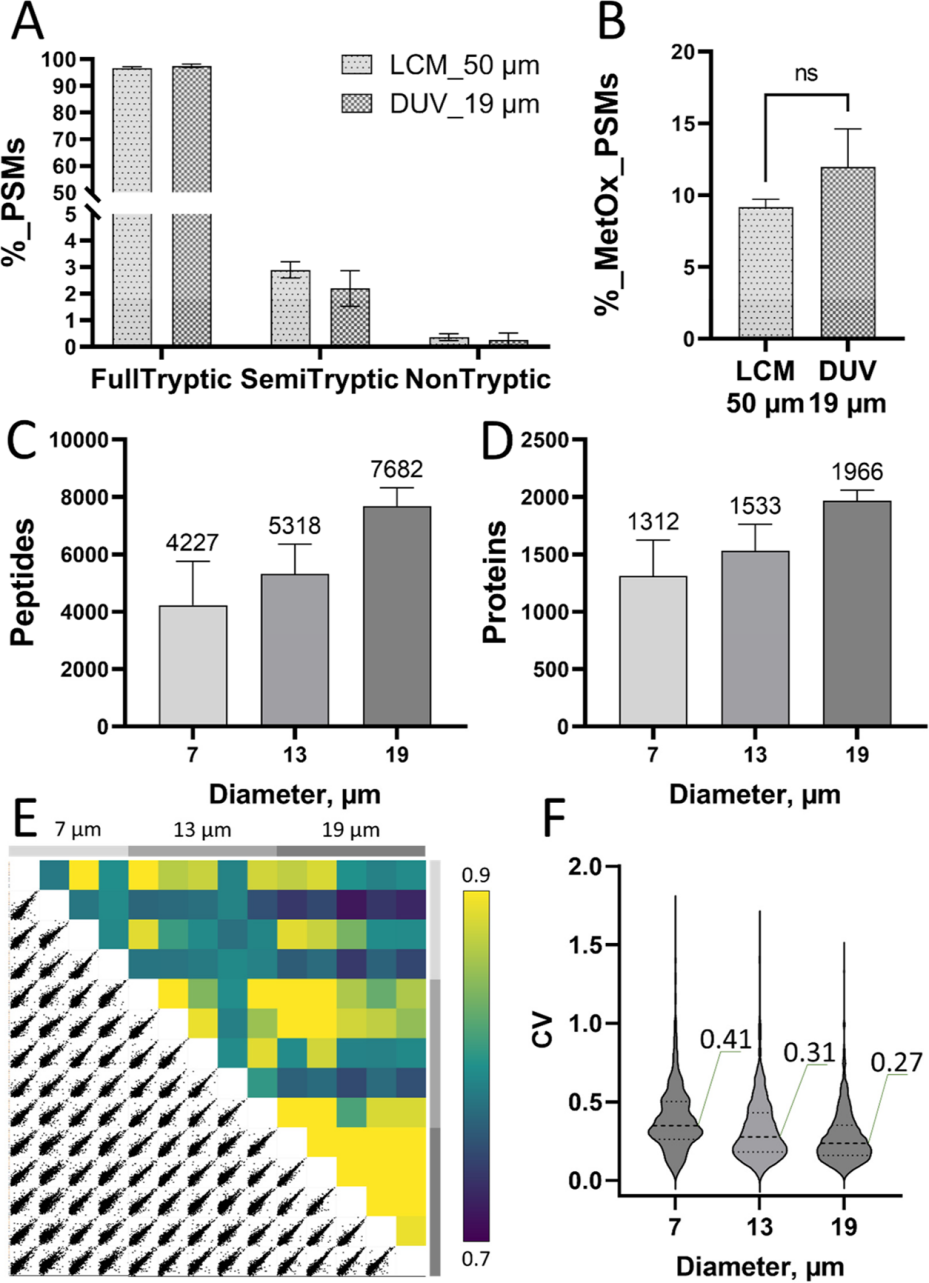
(A) Percentage of peptide-spectrum matches (PSMs) for full-tryptic, semitryptic, and nontryptic peptides and (B) Percentage of PSMs for methionine-oxidized peptides from LCM samples (square cut of 50 μm × 50 μm) and DUV-LA samples (spot size of 19 μm in diameter). Error bars in A and B indicate standard deviation with replicate numbers of 3 for LCM samples and 5 for DUV samples. (C) Unique peptides and (D) protein groups identified from the DUV-LA sample with ablation diameters of 7, 13, and 19 μm. (E) Pairwise correlation of (log-2)-transformed LFQ intensities and (F) Violin plots indicating the distributions of coefficients of variation of protein LFQ intensities for 7, 13, and 19 μm, respectively.

One concern of using the DUV laser for tissue ablation is the potential protein fragmentation or oxidation. Previous DUV laser sampling study^33,34^ demonstrated no observable difference in protein masses between laser-ablated and directly infused protein samples. However, there is growing interest in developing ultraviolet photodissociation (UVPD) approaches for peptide and protein sequencing, indicating that UV-induced fragmentation may be energy-dependent. Because UV-induced dissociation is not specific to certain amino acids (e.g., lysine and arginine), we used a semitryptic rule to search MS raw data from both DUV-LA and LCM isolated tissue samples to evaluate potential protein fragmentation. As shown in Figure 3A, there is no significant difference in the number of semitryptic or nontryptic peptides, indicating the DUV laser-induced protein fragmentation is negligible at the chosen energy settings. It is also well-known that DUV light can efficiently generate ozone in ambient air, which may oxidize proteins during the LA process. To assess this, we compared the percentage of methionine-oxidized peptides in DUV-LA versus LCM isolated tissue samples. As shown in Figure 3B, we did not observe significantly increased oxidization levels in DUV-LA samples. We attributed this to the ultrashort LA process (∼ms per sample), resulting in minimal exposure of the protein samples to ozone.

### Proteome Coverage versus Spatial Resolution

Next, we evaluated the proteome coverage at different LA spot sizes. By changing pinhole sizes, we achieved three different spot diameters: 7, 13, and 19 μm on the acinar region of a 10 μm thick human pancreas tissue section. Each nanoPOTS well-received aerosol particles from a single-spot ablation. As shown in Figure 3C,D, we were able to identify an average of 4227, 5318, and 7682 unique peptides, corresponding to 1312, 1533, and 1966 protein groups from ablation spots with a diameter of 7, 13, and 19 μm, respectively.

To assess the quantitative performance of the DUV-LA-nanoPOTS-based spatial proteomics platform at different spatial resolutions, we performed a pairwise Pearson correlation analysis using the median-normalized protein intensities (Figure 3E). As expected, larger tissue spots exhibited higher correlation coefficients, ranging from 0.90 to 0.94 for 19 μm samples and from 0.85 to 0.91 for 13 μm samples. The correlation coefficients for the smallest tissue spots (7 μm) were lower, ranging from 0.77 to 0.90; this can be attributed to the higher tissue heterogeneities at a subcellular scale. We also calculated the coefficient of variations (CVs) for the three sample groups. For CV calculations, we removed the proteins with >50% missing data in each group. As shown in Figure 3F, similar to the correlation analysis above, lower CVs were observed for larger-sized tissue spots. The median CV values were 0.48, 0.36, and 0.28 for 7, 13, and 19 μm spot sizes, respectively.

### Feature-Specific Proteomes at Subcellular Resolution

**Figure 4:**
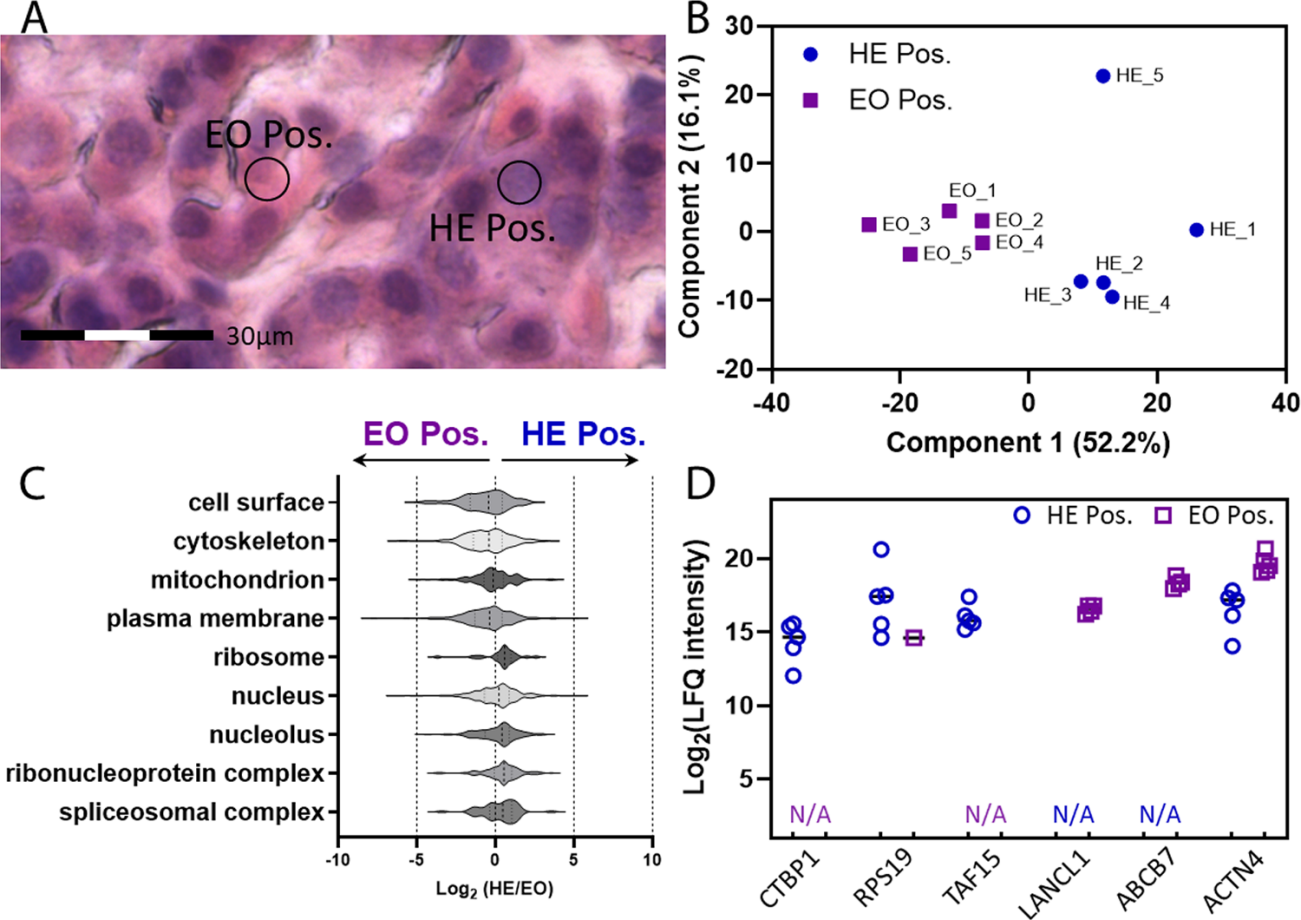
(A) Representative image showing the sample selection for hematoxylin-positive (HE Pos) and eosin-positive (EO Pos) samples. (B) Principal component analysis (PCA) clustering using protein abundance data from HE Pos and EO Pos spots. (C) Relative protein levels (*x*-axis) of major cellular compartments and (D) selected differentially abundant proteins between HE Pos sample spots and EO Pos samples. All samples were isolated with a 7 μm diameter from a 10 μm thick human pancreas tissue section.

In a proof-of-concept study, we selected two distinct regions inside single cells including hematoxylin-positive (purplish blue) and eosin-positive (pink) regions. It is known that hematoxylin stains genetic material in cell nuclei and ribosomes, while eosin stains cytoplasm and extracellular matrix. Samplers were collected based on the H&E image and each tissue sample was collected from a 7 μm spot (Figure 4A). The two sample groups can be clearly segmented based on unsupervised PCA projection (Figure 4B), indicating differential protein abundances in hematoxylin-positive and eosin-positive regions. As expected, higher-abundance proteins in hematoxylin-positive spots were enriched in the ribosome, nucleus, nucleolus, and spliceosome, while proteins in eosin-positive regions were enriched in the cell surface, cytoskeleton, mitochondrion, and plasma membrane (Figure 4C).

As a part of Human Protein Atlas (HPA), Thul et al.^39^ used immunofluorescence microscopy to map the whole human proteome and identify proteins specifically enriched in different cellular compartments. We extracted a list of cytoplasmic and nuclear proteins suggested by HPA and plotted the corresponding protein intensities from our data sets (Figure 4D). For example, CTBP-1 (C-terminal-binding protein 1) is a known corepressor targeting diverse transcription regulators and is heavily enriched in the nucleoplasm of many cell types including HeLa, A-431, U251MG, and U2OS. RPS19, which is the ribosomal protein S19, was classified as localizing nuclear proteins across different cell lines. TATA-box binding protein associated factor 15 (TAF15) is an RNA and ssDNA-binding protein and a typical nucleus marker. All three proteins were exclusively detected in hematoxylin-positive spots in our data sets. In contrast, lanC-like glutathione S-transferase 1 (LANCL1) and actinin α 4 (ACTN4), major components of structural proteins, were highly enriched in the cytoplasm (eosin-positive region). ABCB7, ATP binding cassette subfamily B member 7, a key mitochondrial protein for cellular iron homeostasis, mitochondrial function, and heme biosynthesis,^40^ was exclusively detected in the eosin-positive region. Together, the proof-of-concept study demonstrated the feasibility of subcellular proteomics using a DUV-LA-nanoPOTS-based spatial proteomics platform.

## Conclusions

We developed a high-resolution spatial proteomics platform by coupling DUV-LA with nanoPOTS-based proteomic sample preparation. We systematically optimized the optical setup of the deep-UV LA system and achieved a 2 μm sampling resolution with high consistency. To improve the collection efficiency of aerosolized tissue samples, we placed a DMSO droplet in proximity to the LA spot. This setup enabled the identification of ∼1300 proteins from a 7 μm diameter LA spot, in which the protein content is equivalent to 1/4 of a single cell (∼15 μm diameter). We also demonstrated that subcellular proteomics could be performed with this novel platform to reveal compartment- or organelle-specific functional differences.

Compared with commonly used LCM approaches, our DUV-LA platform has several benefits when applied to spatial proteomics. First, the sample isolation throughput is 5–10 times higher with the single-step DUV-LA process. Up to 30 samples could be collected in 1 min, thus enabling unbiased mapping of the entire tissue section at cellular resolution.^24^ Second, the spatial resolution is improved to ∼2 μm, a resolution that enables the study of the organelle-specific proteomes and protein trafficking in a systematic way. Additionally, the DUV-LA process generates nanometer- and micrometer-sized protein particles, which would improve protein extraction efficiency, especially for formalin-fixed paraffin-embedded (FFPE) tissue samples. Finally, high-quality tissue images can be readily obtained because DUV-LA does not use polymer membrane-coated slides, which allows the seamless integration of digital pathology with deep spatial proteomics.

DUV-LA-nanoPOTS platform offers the ability to perform large-scale proteome mapping of tissue sections for clinical and translational research. However, several challenges remain, particularly the limited throughput of LC-MS-based proteomics. As parallel proteomic sample preparation could be achieved using nested nanowell designs,^41^ future work will be focused on the integration of spatial proteomics platform with sample multiplexing approaches such as isobaric,^42,43^ nonisobaric tags,^44^ or combined precursor isotopic labeling and isobaric tagging (cPILOT).^45,46^

## Supporting Information Available

Rendered image showing camera setup for measuring collection distance (Figure S1). Screenshot of the prototype LabView program for controlling the sample collection process (Figure S2). Procedures for the alignment of the nanoPOTS chip (Figure S3) and delta Z (Figure S4). An example of a stitched image with a 3 × 3 grid (Figure S5). Square ablation holes on the pancreas when a square pinhole was used (Figure S6). Illustration of DMSO splashing phenomena during sample collection (Figure S7). Images showing DMSO splashed on the sample slide (Figure S8) (PDF)
