# Supplementary material for: Spatial Proteomics toward Subcellular Resolution by Coupling Deep Ultraviolet Laser Ablation with Nanodroplet Sample Preparation
Source: ACS Meas Sci Au. 2023 Oct 20;3(6):459–68. doi: 10.1021/acsmeasuresciau.3c00033 (PMC10740121; doi:10.1021/acsmeasuresciau.3c00033)
Supplement: Supplementary file 1 — tg3c00033_si_001.pdf [file tg3c00033_si_001.pdf]

Supporting Information

Spatial Proteomics towards Subcellular Resolution by Coupling Deep Ultraviolet Laser Ablation with Nanodroplet Sample Preparation

Piliang Xiang,[1] Andrey Liyu,[1] Yumi Kwon,[1] Dehong Hu,[1] Sarah M Williams,[1] Dušan Veličković,[1] Lye Meng Markillie,[1] William B. Chrisler,[2] Ljiljana Paša-Tolić,[1,*] and Ying Zhu[3,*]

[1]Environmental Molecular Sciences Laboratory, Pacific Northwest National Laboratory, Richland, Washington 99354, United States

[2]Biological Sciences Division, Pacific Northwest National Laboratory, Richland, WA 99354, United States

[3]Department of Microchemistry, Lipidomics and Next Generation Sequencing, Genentech, 1 DNA Way, South San Francisco, California 94080, United States

[*]Corresponding author:

Dr. Ying Zhu, Email: zhu.ying@gene.com

Dr. Ljiljana Paša-Tolić, Email: ljiljana.pasatolic@pnnl.gov

Contents

Fig. S1. Closeup rendered images showing the LED light (blue arrows) and the Dino-Lite camera for measuring the collection distances (blue arrows) 2

Fig. S2. Screenshot of the prototype LabView program for controlling the sample collection process. 3

Fig. S3. Schematic illustration of the alignment procedures for nanoPOTS chip. 4

Fig. S4. Schematic illustration of the alignment procedures for adjusting the collection distance (chip Z-delta) between nanoPOTS chip and tissue slide. 5

Fig. S5. The image acquisition sequence of the 3 × 3 grid on the left and a stitched pancreas image on the right. 6

Fig. S6. Square ablation holes on pancreas when a square pinhole was used. 7

Fig. S7. The schematic illustration of the DMSO splashing phenomena during sample collection.............. 8

Fig. S8. Images taken immediately after ablation on pancreas using different laser energy levels. 9

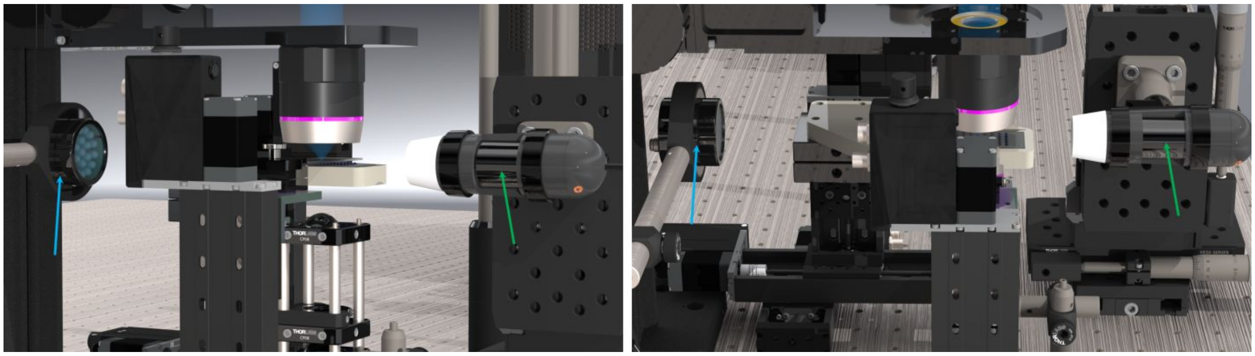

Fig. S1. Close-up rendered images showing the LED light (blue arrows) and the Dino-Lite camera for measuring the collection distances (blue arrows)

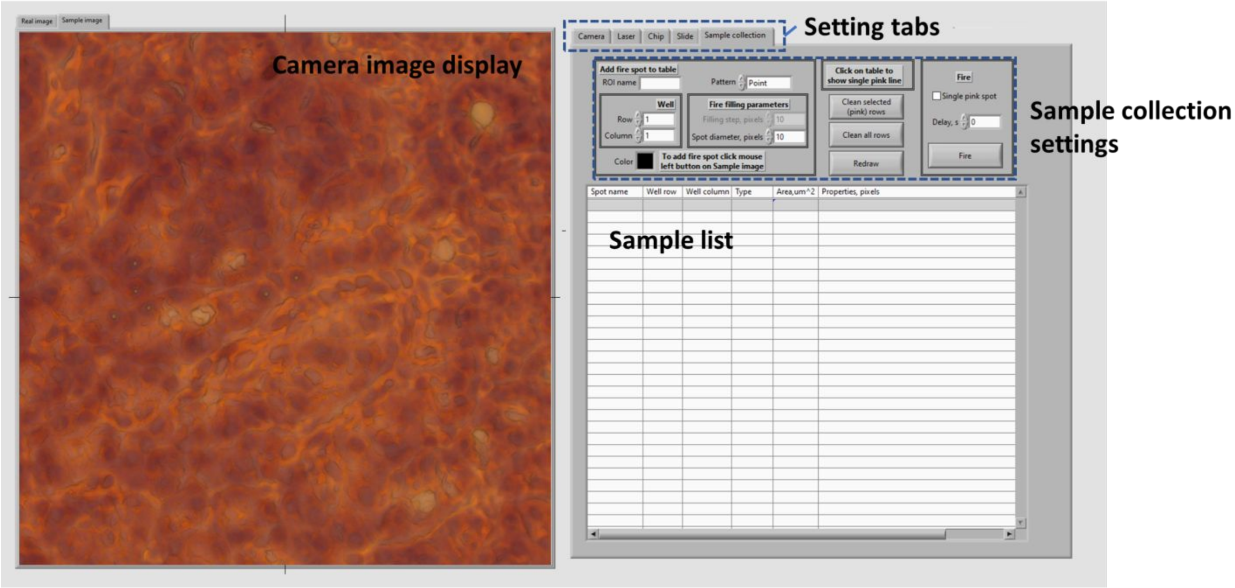

Fig. S2. Screenshot of the prototype LabView program for controlling the sample collection process.

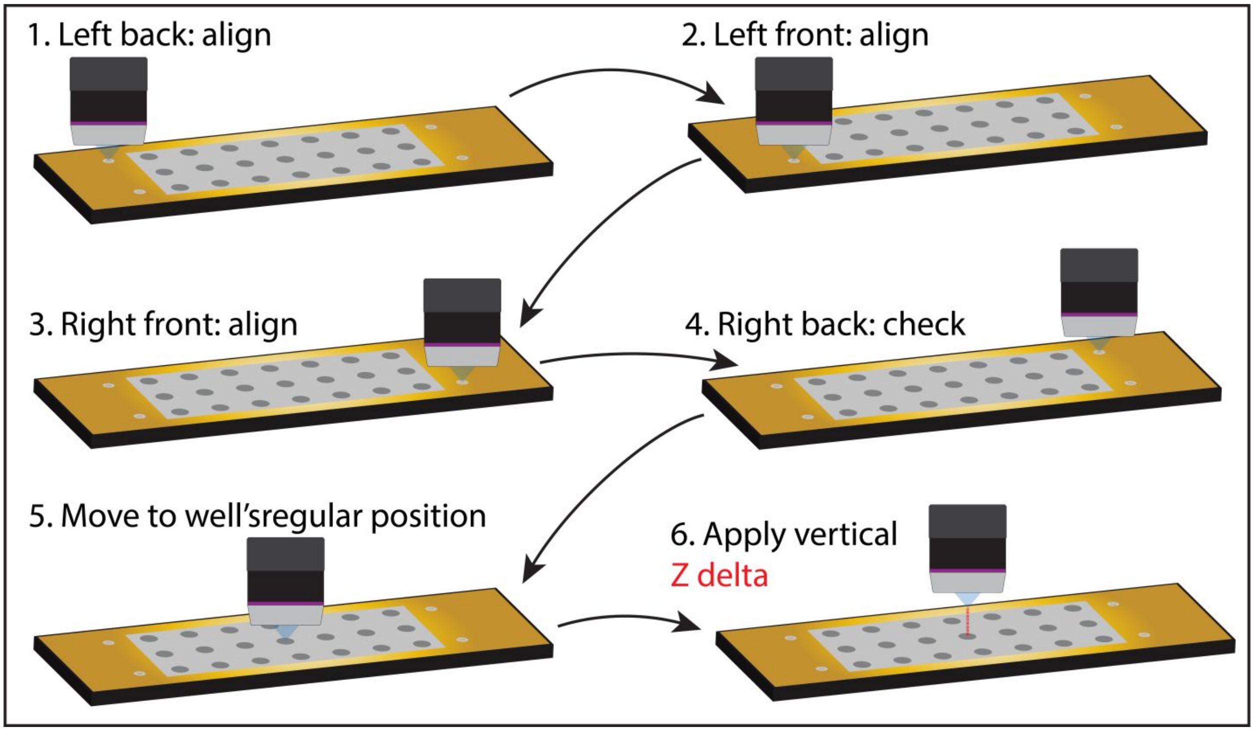

Fig. S3. Schematic illustration of the alignment procedures for nanoPOTS chip.

The chip stage robot was moved until the top left alignment spot is clearly visible in the camera frame at a target position. The stage location information was recorded in the software. The same procedure was repeated for left front and right front alignment spots. The right back alignment spot was used for checking the alignment. All well’s locations can be calculated with the stage location information from the first three alignment spots.

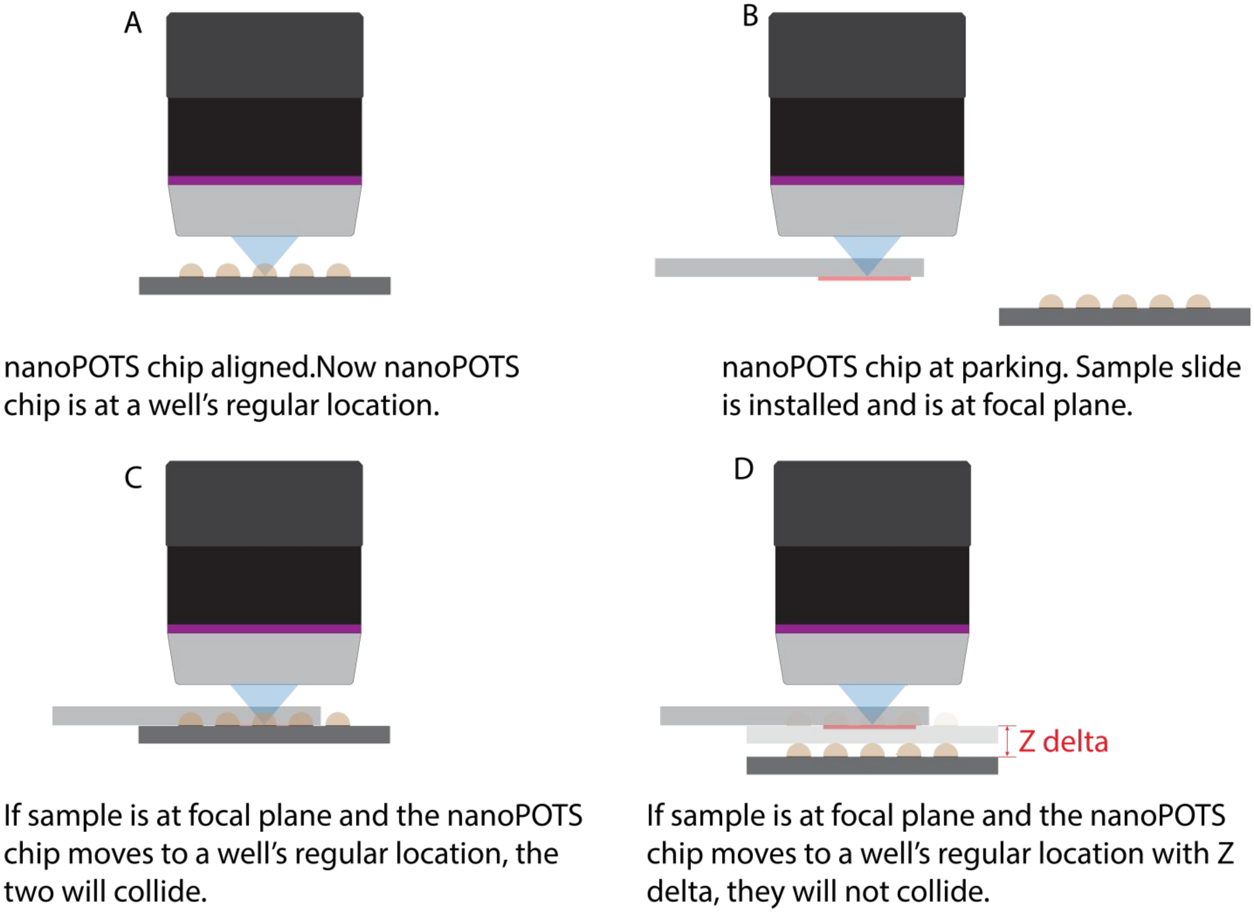

Fig. S4. Schematic illustration of the alignment procedures for adjusting the collection distance (chip Z-delta) between nanoPOTS chip and tissue slide.

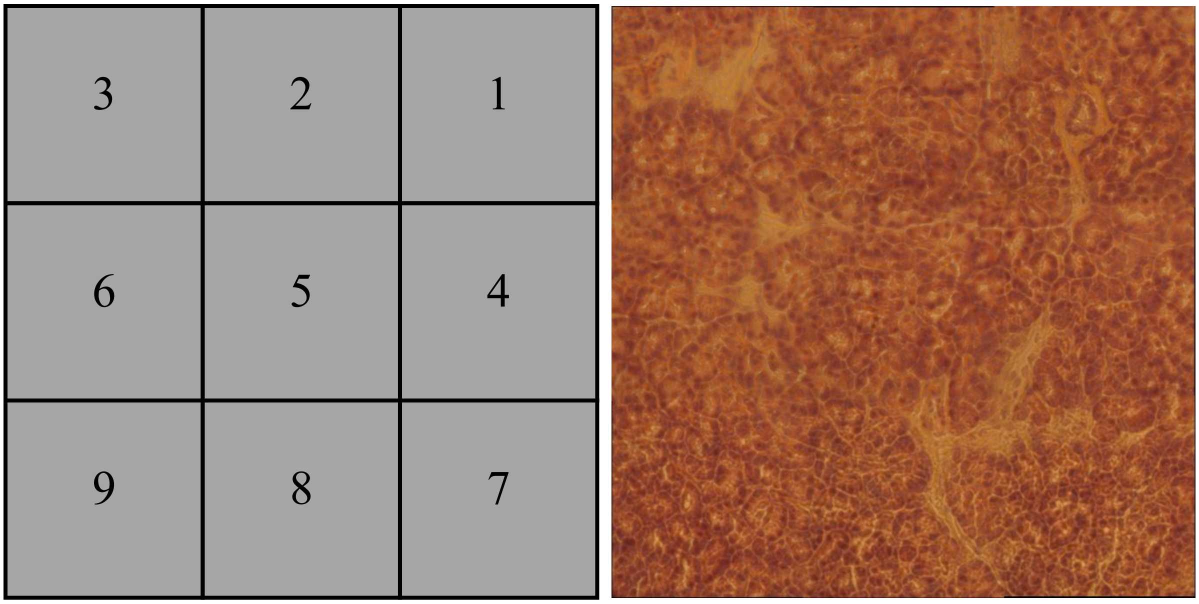

Fig. S5. The image acquisition sequence of the 3 × 3 grid on the left and a stitched pancreas image on the right.

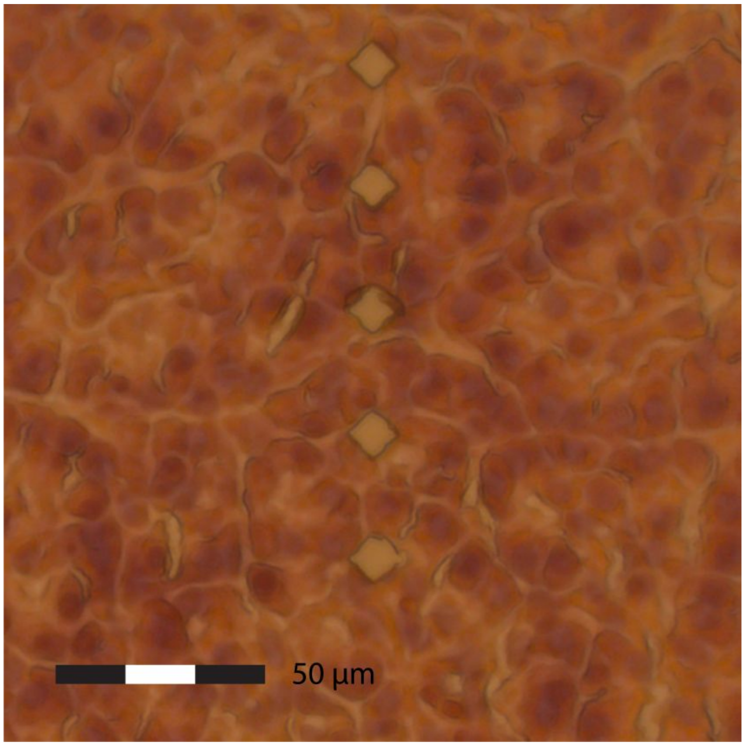

Fig. S6. Square ablation holes on pancreas when a square pinhole was used.

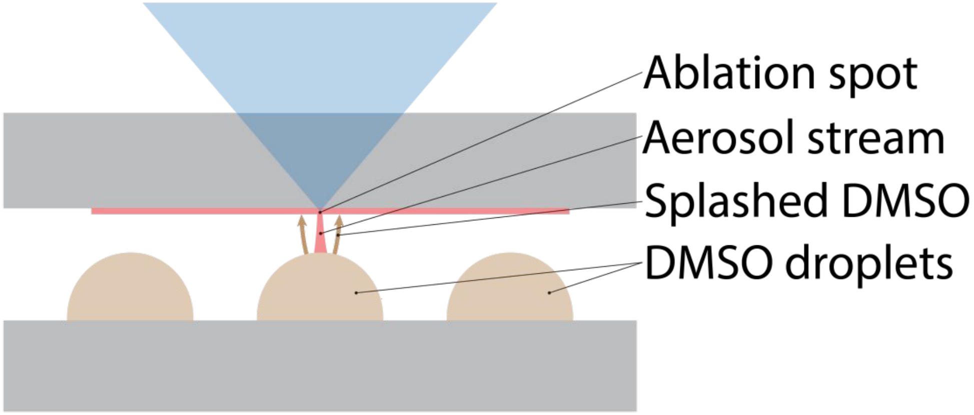

Fig. S7. The schematic illustration of the DMSO splashing phenomena during sample collection. High velocity aerosol particles generated by laser ablation hits a DMSO droplet. Due to the close proximity, the contact velocity of the aerosol on the DMSO droplet is high enough to splash DMSO which is visible under the microscope (in the following Figure S8).

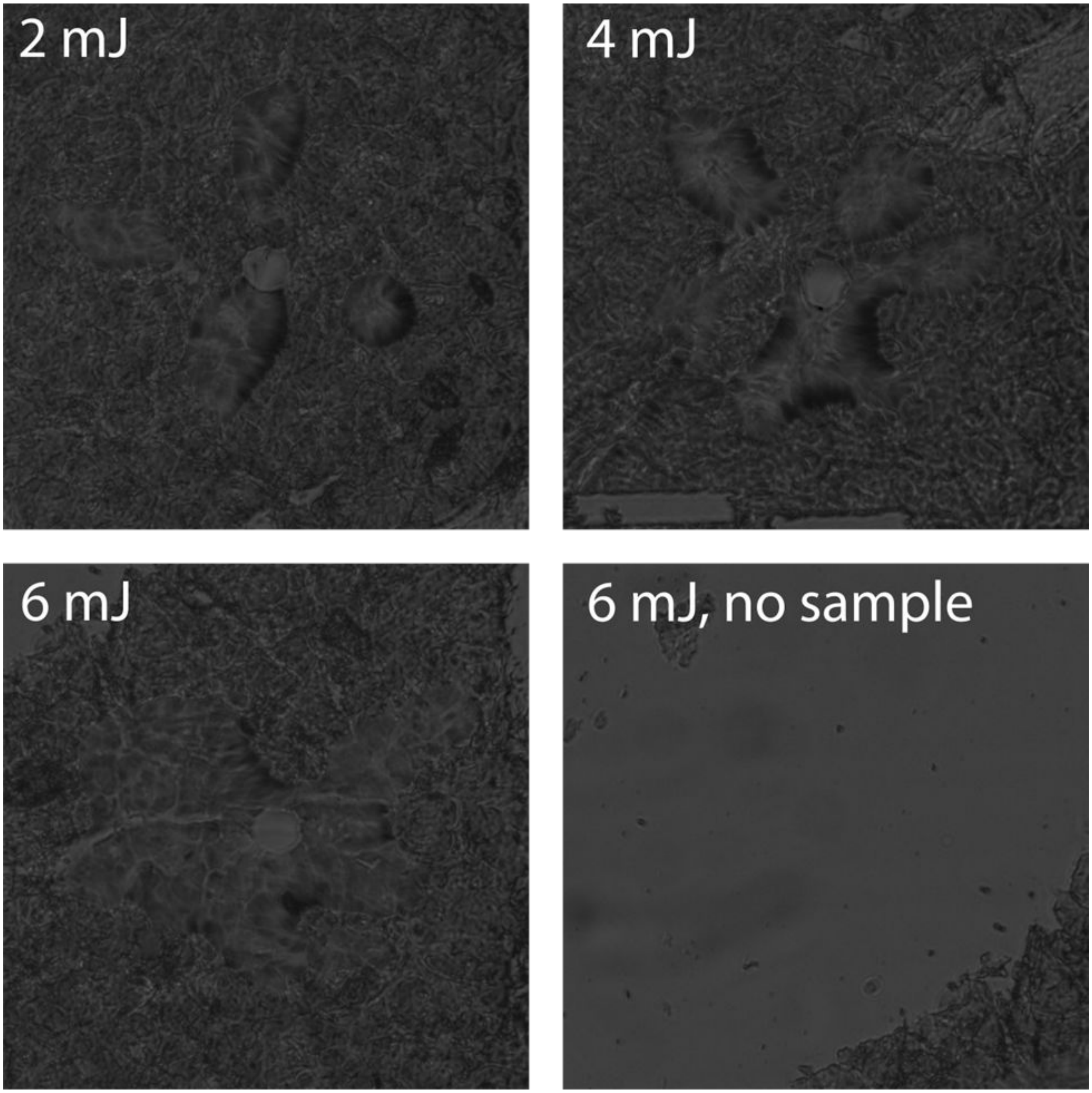

Fig. S8. Images taken immediately after ablation on pancreas using different laser energy levels. Each image is 0.3 mm × 0.3 mm The collection distance was 0.1 mm for all. The energy was set on the laser control software. Higher laser energy (6 mJ) led to more severe DMSO splashing. To confirm that the DMSO splashing was not caused by the laser beam itself, we performed ablation on an area without pancreas sample (6 mJ, no sample). No DMSO splashing visible without pancreas sample.
